# Viewpoint: Difficult-to-treat depression versus treatment-resistant depression: A new integrative perspective for managing depression

**DOI:** 10.1192/j.eurpsy.2023.2448

**Published:** 2023-09-08

**Authors:** Walter Paganin

**Affiliations:** PhD Student in Neuroscience, University of Roma Tor Vergata, Rome, Italy; Vocational Training in Advanced Clinical and Medical Research Methodologies, University of Bologna Alma Mater Studiorum, Bologna, Italy

**Keywords:** clinical management, difficult-to-treat depression, multidisciplinary, treatment-resistant depression

## Abstract

In the STAR*D study, the efficacy of treatments for major depression was examined. It was found that, while many responded to the initial antidepressant treatment, only 30% of participants achieved complete remission. Concerning treatment resistance in depression, there is a recent distinction emerging between treatment-resistant depression (TRD) and difficult-to-treat depression (DTD). Historically, TRD and DTD have been conflated, but it is essential to recognize them as separate entities. While TRD is characterized by a patient’s inadequate response to two or more consecutive antidepressant treatments given for an adequate duration and dosage without achieving acceptable therapeutic effects, DTD describes a clinical category where patients do not achieve full symptom control despite various therapeutic approaches. The recent shift in perspective proposes a more integrated approach for DTD, encompassing psychosocial, biological, and interactive factors. This multifactorial model calls for a multidisciplinary therapeutic intervention, not restricted to pharmacological treatments but also including psychotherapy, neurostimulation, and social interventions. Informing professionals and the general public about the significance of this new approach could mitigate the stigma associated with depression and enhance the quality of care. The future challenge will involve a deeper clinical understanding of DTD and its optimal management by refining available treatments.

The STAR*D study, sponsored by the National Institute of Mental Health (NIMH), investigated the efficacy of treatments for major depressive disorder in individuals unresponsive to initial antidepressant therapy. It was found that approximately 50% of participants (aged 18–75 years) responded to an initial antidepressant regimen, while only 30% achieved symptomatic remission. Some showed a positive response to secondary antidepressant therapies or a combination of two medications, yet others remained unresponsive to any treatment regimen for a duration of at least 12 months. A third of the cohort failed to achieve full recovery even after multiple pharmacological interventions, demonstrating resistance to pharmacotherapy [1].

Treatment resistance in depression poses a significant challenge for clinicians and researchers dealing with patients suffering from depression, particularly in *difficult-to-treat depression* (DTD), where a clinical condition of depression is identified, which, despite standard therapeutic efforts, does not demonstrate full symptom control. This places a substantial burden on the patient, their family, and the clinical practitioners involved in their care [2]. This condition significantly impacts patients’ quality of life, elevating the risk of disability, suicide, and other complications [2]. DTD should not be confused with *treatment-resistant depression* (TRD), which has long captured the attention of the medical and scientific community. TRD does not represent a new diagnostic category but rather a clinical scenario invoked when a patient fails to respond adequately to two or more antidepressant treatments at appropriate dosages administered consecutively over a sufficient period without achieving acceptable therapeutic effects, as recently highlighted by the *European Medicines Agency* [3]. Over the years, TRD has been the subject of numerous classifications and research efforts aimed at identifying new pharmacological therapeutic strategies for resistant patients. The very definition of TRD has been a topic of debate among experts, as there is no shared consensus on the criteria that determine treatment nonresponse [4]. Historically, terms such as TRD and DTD have been used interchangeably and synonymously, causing considerable confusion in the scientific literature. These two clinical conditions represent distinct forms of depressive disorders that should be clearly recognized and differentiated [5, 6]. Already, 20 years ago at a symposium titled “Difficult-to-Treat Depression,” the idea was proposed that DTD was a more accurate label than TRD [7].

Recent developments and research have led to a broader and more inclusive approach to DTD, emphasizing the need to consider a concept that goes beyond mere resistance to pharmacological treatments. This new perspective, encompassing psychosocial, biological, and interactive aspects, paves the way for an integrative model of therapeutic management of resistance in depression [5]. DTD is not a binary condition, but rather exists along a therapeutic response continuum. This spectrum encompasses complete responses, partial responses, and even nonresponses, shifting the treatment focus from a curative/remissive model to a disease management model that emphasizes enhancing functionality and quality of life, aiming for optimal symptom control [8, 9]. An international consensus, comprising 15 academics from across Europe, the United States, Canada, and Australia, with expertise in affective disorders, was established in 2020 [2] and has proposes a cultural and scientific shift to guide clinicians and researchers, expanding the TRD model [10]. While TRD is associated with the inefficacy of standard pharmacological treatments, DTD encompasses a broader clinical perspective that incorporates psychological, social, environmental, and patient care system interactions that can influence treatment response. To identify patients with DTD, it is essential to consider the course of depression, symptom variability, notably the presence of anhedonia and anxiety, functional impairment, coexisting psychiatric or general medical conditions, including substance use disorders, and considerations of concurrent medications. The patient’s medical history should highlight the number and sequence of treatments undertaken, types and counts of therapeutic failures, familial history, treatment adherence, and the presence of childhood traumas [8]. Thus, the key distinctions between TRD and DTD pertain to the very definition of the clinical condition and the therapeutic approach. The definition of DTD represents a significant cultural shift, grounded in both clinical practice and treatment, underscoring the complexity of the condition and the importance of considering multiple variables at play [5, 10].

Theorizing the DTD model’s study, it is crucial to emphasize the importance of altering the methodological approach from what was used previously. Such changes should encompass the integration of diverse operational criteria, which factor in the consensus among various experts in the research group. It is also vital to incorporate individuals who have personally experienced depression, termed as people with lived depression (PWLD), into this group. Moreover, there is a pressing need to set clear and unequivocal operational standards to pinpoint crucial elements that demand explicit definitions. These elements encompass the count of antidepressant treatments, inclusion/exclusion guidelines, validation of relevant psychometric and assessment tools, traits of pharmacotherapy, outcomes, and the assessment of the efficacy of novel therapies [6]. As far as DTD is concerned, it is desirable to be able to deepen the recognition of potential biomarkers and evaluate the neurobiological facets linked to the various clinical manifestations of DTD [11]. Summarizing the 2020 international consensus on DTD management, the main focus should be on addressing depressive symptoms while equipping patients with coping tools. The goal of treatment requires a multidisciplinary approach that goes beyond the simple evaluation of pharmacological treatments and should move from aiming solely at remission, improvement of symptom control and quality of life [2], see Table 1.

**Table 1.** 

1	Prioritize conventional treatment modalities, when they are not enough, explore other treatments.
2	Identify symptoms that lead to worse clinical outcomes, for example, anhedonia, anxiety, and pain.
3	Address symptoms that hinder quality of life, such as sleep disturbances, fatigue, and cognitive problems.
4	To minimize symptom severity, address physical health concerns, substance abuse, associated psychiatric conditions, and treatment-induced problems.
5	Ensure consistent monitoring and treatment for best long-term outcomes.
6	Equip patients with self-management tools, which may involve: - Training to counter negative thinking. - Adoption of behavioral activation techniques. - Encourage community involvement. - Emphasizing regular sleep and physical activity. - Highlight nutritional well-being. - Strengthen coping mechanisms. - Recommend role adjustments. - Promote digital tools for managing depression.
7	Offer a supportive environment and formulate personalized treatment plans, emphasizing the role of the patient.
8	Periodically review the diagnosis and treatment strategy, re-evaluate the diagnosis when necessary, and scan for other conditions.

This approach should encompass psychotherapies, neurostimulation techniques, social and occupational interventions, all aimed at enhancing adherence and therapeutic effectiveness. It also focuses on fostering self-management of symptoms and fostering integration among healthcare professionals, patients, families, and the broader community [2].

Another focus should include enhancing individual, family, professional, and social interactions, with an emphasis on harmonizing the therapeutic intentions of clinician and patient. Decisions in treatment ought to consistently integrate patient viewpoints, aligning with the expertise of medical professionals and adapting interventions to address the specific challenges presented by DTD [12]. It is imperative to scrutinize all potential therapeutic avenues, particularly, when the achievement of symptomatic remission remains ambiguous. Family involvement is of paramount importance in the treatment of complicated and difficult depressions as evidenced by multiple academic surveys [10, 13]. The dispositions of families and societal conceptions concerning DTD considerably influence the progression of the disorder [10].

Raising awareness among the general public, specialists, and physicians about the importance of addressing these aspects in the management of DTD can help reduce the stigma associated with TRD and enhance the quality of life for patients. Physicians should become well-acquainted with DTD and its therapeutic options, as a deeper understanding of DTD and its treatment choices can lead to improved patient outcomes. As the scientific community continues to unearth the underlying intricacies, there’s hope that future endeavors will lead to better diagnostic tools, more effective treatments, and a deeper understanding of the disorder. At present, there is not a clear classification or specific taxonomy for DTD. This absence leads to diagnostic uncertainties and hinders clinical research, underscoring the need for further studies and detailed analyses. As a result, it is essential to develop new research methodologies and select appropriate experimental designs to accurately assess causal inferences and the applicability of clinical study findings [14]. In the future, the primary challenges will focus on the management of DTD and the optimization of treatments [15].
